# Autonomic Testing in Functional Gastrointestinal Disorders: Implications of Reproducible Gastrointestinal Complaints during Tilt Table Testing

**DOI:** 10.1155/2009/868496

**Published:** 2009-05-05

**Authors:** Shaista Safder, Thomas C. Chelimsky, Mary Ann O'Riordan, Gisela Chelimsky

**Affiliations:** ^1^Department of Pediatrics, Rainbow Babies and Children Hospital, Case medical Center, 11100 Euclid Avenue, Cleveland, Oh 44106, USA; ^2^Department of Neurology, University Hospitals Case Medical Center, Case Western Reserve University School of Medicine, 11100 Euclid Avenue, Cleveland, Oh 44106, USA

## Abstract

*Background*: The pathophysiology of functional abdominal pain (FAP) is unknown. The upright portion of a tilt table test triggers typical symptoms in certain children. Aim: To compare the pathophysiology and treatment response of children with FAP whose gastrointestinal symptoms (GI) were replicated (RGI) by tilt table testing (TTT) to those in whom TTT did not have this effect (NRGI). *Methods*: An IRB-approved retrospective review of the autonomic laboratory database identified all children tested for GI complaints. We compared results of TTT, Valsalva maneuver, deep breathing and the axon reflex sweat test. Overall treatment response and that specific to fludrocortisone was ranked from 1 to 5, with 1 “much worse,” 3 “neutral,” and 5 “much better.” *Results*: 32/76 identified children had reproducible symptoms on TTT (RGI) and 44 did not (NRGI). The RGI group was younger, had a shorter duration of symptoms, more postural tachycardia syndrome (POTS) and benefited more from fludrocortisone (73% in RGI vs. 25% in NRGI). *Conclusion*: Dividing patients with FAP according to the effect of TTT on their symptoms appears to delineate 2 fundamentally different groups, with potentially different pathophysiologies and treatment responses. A prospective study is needed.

## 1. Introduction

Functional gastrointestinal disorders (FGID) affect a large percent of children and effects quality of life. The exact prevalence of FGID in children is unknown. One study found 10–20% of children suffer from chronic abdominal pain [1]. Also, 6% of middle school students and 15% of high school students fulfill the criteria for irritable bowel syndrome [2]. These children have a decreased quality of life with increased school absenteeism, decreased play time, and other significant impairments.

According to the proposed biopsychosocial conceptualization of functional gastrointestinal disorders, the autonomic nervous system links the psychosocial factors with the pathophysiological elements [3]. A role for abnormal autonomic nervous system function is increasingly recognized which may not be limited to the gastrointestinal tract. In pediatrics, exploratory studies have found autonomic dysregulation in pediatric FGID [4–6]. FGID's have been associated with orthostatic intolerance (OI), mainly postural tachycardia syndrome (POTS) and syncope [4, 6], but other associations have not been carefully evaluated. Three main types of orthostatic disorders have been described in patients undergoing tilt table testing (TTT): postural tachycardia syndrome (POTS), reflex syncope, and orthostatic hypotension (OH).

 POTS is defined as an increase in heart rate (HR) from supine to standing position of greater than 30 bpm, without sustained drop in blood pressure. This definition has a stronger clinical implication if the increase in heart rate is associated with symptoms replicating the chief complaint for the testing, like dizziness, headache, nausea, and so forth. The symptoms of POTS and many of the symptoms of functional gastrointestinal disorders, particularly dyspepsia, overlap. When Sandroni et al. [7] described the symptoms associated with POTS including dizziness, lightheadedness, and lower extremity weakness, they also reported frequent associated symptoms of dyspepsia, such as nausea, bloating, early satiety, and abdominal pain. In addition, patients with POTS who have an associated autonomic neuropathy harbor more gastrointestinal symptoms than those who do not [5].

This association also holds true when explored from the reverse, gastrointestinal perspective. Children with chief complaints of abdominal pain and diagnosed with functional gastrointestinal disorders have dizziness (58%), headaches (52%), while 10% have prominent fatigue, and 5% have episodes of loss of consciousness [8–10]. Sullivan et al. [6], also described similar findings in children with functional gastrointestinal disorders, with lightheadedness and fatigue in 46%, however this study was limited by a small sample size and a skewed population that was sampled .

We have frequently observed the reproduction of gastrointestinal symptoms when the subject is upright during the head-up tilt table test. The clinical implications and associated outcome of such findings have not been evaluated. The purpose of this study was to determine, through a review of comprehensive autonomic testing performed in children with functional gastrointestinal disorders if replication of symptoms during tilt test is associated with a different treatment outcome than when symptoms are not present during TTT.

## 2. Material and Methods

### 2.1. Study Design

A retrospective University Hospitals Case Medical Center Institutional Review Board approved chart review was conducted from the Pediatric Autonomic Laboratory database containing 419 pediatric records between 1994 and 2007. From this group, we selected only those children referred for gastrointestinal (GI) complaints (*n* = 76). We excluded children with a known organic gastrointestinal disease or genetic/metabolic abnormality. Most of the children had been referred to the Autonomic Clinic by their pediatric gastroenterologist or general pediatricians for evaluation of dysautonomia as a possible cause of their gastrointestinal complaints.

### 2.2. Testing

All patients underwent 4 tests of autonomic function including 3 tests of cardiovascular function (the cardiovascular responses to deep breathing, to the Valsalva maneuver, and to tilt table testing) and one test of sweating, the sudomotor axon reflex response. The patients stopped any medications that could affect autonomic function for 5 half-lives prior to testing, such as beta-blockers, caffeine, anticholinergic medications, and so forth.

 TTT consisted of 20-minute supine rest on a motorized tilt table prior to an upright tilt to 70°. Continuous BP and HR were monitored noninvasively in the supine position for 3 minutes, and upright for 10 to 40 minutes. The diastolic BP normally decreases less than 10 mmHg, and the systolic BP, less than 20 mmHg, and HR should increase less than 30 bpm [9].

Deep breathing consists of 6 deep breaths per minute, with the HR variation calculated from the best 6 of 12 cycles (normal values in this age group > 22 bpm [11]). Results are reported as the average difference between inspiration and expiration of the 5 best breaths. For the Valsalva maneuver, the subject maintains a 40 mmHg pressure gradient with an open glottis for 15 seconds, while HR and blood pressure (BP) are continuously monitored. The Valsalva ratio consists of the fastest HR during pressure exertion (termed phase II, sympathetically mediated) divided by the lowest HR after pressure release (termed phase IV, parasympathetically mediated). Normal values depend on age [11].

Sudomotor axon reflex response to the iontophoresis of acetylcholine (10% with a 2 mA current for 5 minutes, recording sweat output for 10 minutes, “QSART”) across the skin of the feet, calves, hands and upper arms, was measured by standard methods [12]. Normal values vary by body site and gender, and were not considered abnormal unless abnormal axon reflex response found in 2 sites were outside of the range of normal, defining the presence of an autonomic neuropathy.

### 2.3. Data Analysis

Patients were divided into two groups on the basis of their symptoms during the TTT: patients with reproducible GI complaints (RGI) and those without (NRGI). The response to treatment was scored from 1 to 5 with 1 “much worse,” 2 “little worse” 3 “neither better nor worse,” 4 “somewhat better” and 5 “much better.” The score of 5 was reserved for patients whose symptoms resolved completely. For analysis purposes, subjects with a “somewhat better” response (rating of 4) and those with a “much better” (response rating of 5) were collapsed into a single group and compared to all others. Thus a favorable treatment response was ≥ 4 out of 5 on the symptom response scale.

Three orthostatic disorders were defined for this group of patients: POTS required an increase in (HR) of greater or equal to 30 bpm in the first 10 minutes of upright position during tilt test associated with symptoms, and without a drop in blood pressure. While the adult definition also accepts an absolute heart rate of 120 bpm we did not include this criterion in this population, since 120 bpm may be represent a normal (HR) in younger children. Reflex syncope required an abrupt drop in blood pressure and often (HR) (over 3 minutes or less) leading to syncope or pre-syncope. OH was defined by a sustained drop in blood pressure or ≥ 20 mmHg systolic or ≥ 10 mmHg diastolic in the first 3 minutes of the tilt study. Patients with an orthostatic disorder who also had syncope were classified in the group with the orthostatic disorder, as this was considered to be the underlying process. For example, if a patient had POTS and syncope, they were placed in the POTS group.

The study population was described overall and by group. Nominal variables were described by frequencies and percents. Continuous variables were described using the appropriate measures of central tendency and dispersion. Groups were compared using two-sample tests of significance. Nominal variables were compared using Fisher's exact test, and continuous variables were compared using either a two-sided *t* test, or Wilcoxon rank sum test, depending on the distribution. All analyses were done using SAS, v 9.1 (the SAS Institute, Carey, NC). The level of significance was set at 0.05.

## 3. Results

**Figure 1:**
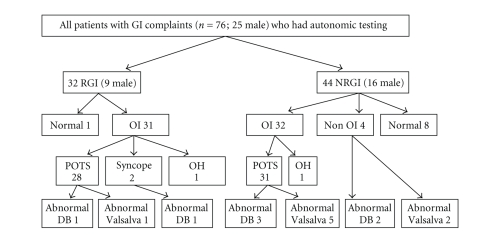
Result of Autonomic testing. OI: orthostatic intolerance, DB: Deep breathing, and OH: orthostatic hypotension.

Of the 76 children included in the study, 51 were females with a mean age of 13.4 ± 3.6 years. In 32 out of 76 children the tilt table test replicated their GI complaints (RGI) while in 44 it did not (NRGI). Table 1 characterizes the two groups and Figure 1 describes the results of autonomic testing. The RGI group was younger and had a shorter duration of symptoms. Abdominal pain was the most frequent chief complaint in the RGI group, though some subjects had more than one chief complaint. The predominant complaints in each group are summarized in Table 2, with no difference between groups (*P* = .17).

The symptom replicated during tilt was abdominal pain in 28/32 (87%) of the RGI group, nausea 2/32 (6%) and vomiting in 2/32 (6%). By definition, symptoms were not replicated in the NRGI group (Figure 3). The RGI group had a greater frequency of POTS than the NRGI group (88%) 28/32 versus (70%) 31/44 (*p* = 0.09, Table 1). Many patients with POTS also had reflex syncope, present in 18/32 (56%) in the RGI group and 10/44 (23%) in the NRGI group. OH occurred in 1 subject in each group. Abnormal cardiac reflexes (abnormal Valsalva maneuver or abnormal deep breathing) were more common in the NRGI group, though this did not reach statistical significance. In the NRGI group, 8/44 children had normal tilt testing test (18%), whereas only 1 patient had normal testing in the RGI group. All but one patient on TTT in the RGI group showed some form of orthostatic intolerance with patients having POTS, syncope, OH or both POTS and syncope. The distribution of abnormalities is rendered algorhythmically in Figure 1. There was no significant difference between quantitative sudomotor axon reflex findings in the two groups (an indicator of autonomic neuropathy).

**Figure 2:**
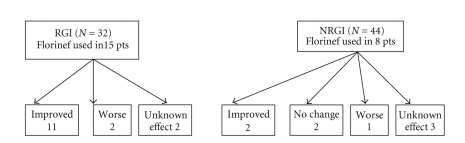
Fludrocortisone treatment effect.

**Figure 3:**
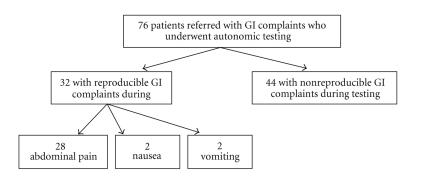
Gastrointestinal (GI) Symptom profile of patients who underwent autonomic testing.

Treatment was not standardized and generally selected based on symptoms and results of the autonomic nervous system testing, often with more than one agent (Table 3). In addition, all patients were asked to supplement salt and fluids to their diet, and increase their physical activity. Not surprisingly, patients in the RGI group were more often treated with fludrocortisone 15/32 (46%) aimed at their orthostatic symptoms (mainly POTS) compared to the NRGI group where only 8/44 (18%) received fludrocortisone *P* = .01. Of patients receiving fludrocortisone, patients in the RGI group tended to benefit more, with 11/15 (73%) reporting an improvement in their symptoms versus 2/8 (25%) in the NRGI group (Figure 2).

## 4. Discussion

This study divided patients with functional abdominal pain into a subgroup whose symptoms were replicated during the upright portion of the tilt table study (RGI) and a subgroup where this did not occur (NRGI). The results suggest that this classification may make sense from both a physiologic and an outcome perspective. The RGI group tended to be younger, with a shorter duration of symptoms, a preponderance of abdominal pain, and a higher incidence of POTS. These children appeared to respond better when treated for their orthostatic symptoms with fludrocortisone. In contrast, the older NRGI subgroup had less POTS, and most of the abnormal autonomic reflexes in the entire cohort were found in this subgroup. From an autonomic perspective, the RGI group was quite homogeneous, as nearly all patients had some form of orthostatic intolerance on TTT (POTS, Syncope, and OH). On the other hand, the NRGI group appears more heterogeneous, with some subjects entirely normal, some with the same findings as the RGI group, and some even more abnormal with abnormal cardiovascular reflexes such as a reduced response to deep breathing or reduced Valsalva ratio. Such a discrepancy between the two groups further suggests that symptom replication by tilt study may be physiologically important.

One could imagine several explanations for the difference between these groups. Both sets of children could be suffering from the same disorder, but the RGI group in whom abdominal pain predominated may have harbored more bothersome symptoms, resulting in an earlier presentation. According to this hypothesis, the disorder might evolve through a phase in which symptoms are more variable depending on patient activity, diet, position, and so forth, (represented by the RGI group in this study) to a final stage in which symptoms become “hard-wired,” here represented by the NRGI group. Support for this notion can be drawn from the chronic pain literature, where such a progression is well recognized, for example, in complex regional pain syndrome, and in chronic failed back syndrome [13, 14].

Alternatively, our findings could reflect 3 physiologically distinct groups of children with functional abdominal pain. The first group (the entire RGI cohort) has symptom replication on tilt table testing, owing to some type of dynamic alteration in gastrointestinal blood flow during orthostatic challenge, an abnormality that is more responsive to medical management, with consequent better outcome. This group might correspond to the term “gastrointestinal vasomotor dysautonomia.” A second group, perhaps termed “gastrointestinal neuropathic dysautonomia” would constitute the NRGI subgroup with abnormal autonomic testing, reflecting a static abnormality of autonomic innervation to the gut, perhaps resulting in abnormal motility, and less responsiveness to treatment. The third group, “gastrointestinal nonautonomic dysfunction” would comprise the NRGI subgroup with entirely normal autonomic testing, in whom there is neither a dynamic blood flow abnormality, nor a static loss of innervation with dysmotility, and where the abdominal pain appears to bear no relation to any autonomic abnormality.

The concept of a gastrointestinal vasomotor disturbance accounting for symptomology in the RGI group dovetails well with some recent POTS investigations, emphasizing mesenteric hyperemia as a major finding in certain POTS subgroups [15]. As postulated by Stewart et al. the mechanism of this hyperemia could include a focal denervation, or more likely, local neuroendocrine factors in the enteric nervous system, such as nitric oxide or vasoactive intestinal peptide resulting in inappropriate vasodilation. Other considerations might include abnormal autonomic-enteric influences, or even central nervous system alterations, as postulated in abdominal migraine. Serotonergic pathways could play a role here, since both POTS [16] and functional abdominal disorders (e.g., IBS [17]) have demonstrated serotonergic abnormalities. Future studies could determine whether there is an association between splanchnic hyperemia and the presence of tilt-induced gastrointestinal symptoms and the potential role of gastrointestinal motility.

Clinically, the classification into 3 groups may have some utility. The “gastrointestinal vasomotor dysautonomia” subgroup is slightly younger, have had symptoms for less time, and respond better to treatment. The “gastrointestinal neuropathic dysautonomia” and “gastrointestinal non-autonomic dysfunction” groups, both contained in the NRGI group in this study, may well differ in characteristics, but will require a larger prospectively gathered cohort for a better comparison of their features and outcomes. Utilizing this putative classification to classify patients prospectively will also likely determine which treatment modalities provide optimal benefit in each group, and further delineate the phenotype of these children.

A majority of patients, regardless of subgroup, demonstrated POTS as the primary abnormality on autonomic testing. The pathophysiology underlying this disorder remains elusive to-date, although, akin to the proposed classification above for functional gastrointestinal disorders, one can group patients into those who have abnormal autonomic reflexes and those who do not [5]. POTS represents a form of nonspecific autonomic dysregulation, which may be classified as resulting from either a peripheral or a central autonomic nervous system etiology, based on the axon reflex response [5]. Interestingly, those with a presumed peripheral cause in this particular study had a greater frequency of migraine headache and gastrointestinal symptoms. However, in the present study we did not find a difference in axon reflex responses, and thus no suggestion that a peripheral autonomic process is more likely in one dysautonomic group than in the other. This could, however, relate to the lack of power in the present study to detect such a difference.

This study has several additional limitations. The large number of children with functional abdominal pain who had POTS (77%, Table 1) could result from a referral bias, since many patients were sent for evaluation of dysautonomia. A randomly selected population of patients with functional gastrointestinal disorders may harbor less POTS. Secondly, since children are being treated clinically without a standardized care-path, treatment benefit is difficult to assess objectively, and we do not truly know the number of patients who improved with medications targeted at various putative mechanisms. For the same reasons, we do not know how many patients benefited from physical activity and other forms of conditioning, which we commonly prescribe. Thirdly, we do not have adequate information about puberty and menstrual status in our patients, to know the significance of this factor in the subgroups. Finally, the study is retrospective, and subject to well-known biases of such a design, as already alluded to earlier in the discussion. Nonetheless, it is fair to summarize by stating that autonomic testing reveals different and interesting subpopulations of patients with functional abdominal disorders, which may harbor physiologic importance, and ultimately lead to a better understanding of the mechanism of these symptoms and their treatment.

## Figures and Tables

**Table 1:** Summary of demographic characteristics and results of autonomic testing in each group.

	Overall *N* = 76	RGI *n* = 32	NRGI *n* = 44	*P* value
Mean age (years) ± SD	13.4 years ± 3.6 yrs	12.6 ± 3.3	14.1 ± 3.70	.08
Male gender	25/76 (32%)	9 /32 (28%)	16/44 (36%)	.46
Median duration of symptoms (range in months)	41.5 ± 24.0	29.3 ± 30.9	51 ± 45.6	.04
	24(1-156)	18(2-120)	35(1-156)	
Abnormal Valsalva Ratio (no. subjects)	8/76 (10%)	1/32 (3%)	7/44 (15%)	.13
Abnormal Deep Breathing (no. subjects)	7/76 (9%)	2/32 (6%)	5/44 (11%)	.69
Either DB and Valsalva ratio abnormal	15/76 (20%)	3/32 (9%)	12/44 (27%)	.13
Abnormal Axon-Reflex Response	23/76 (30%)	10/32 (31%)	13/44 (29%)	.81
POTS on TTT	59/76 (77%)	28/32 (87%)	31/44 (70%)	.09
Patients on Fludrocortisone	23/76 (30%)	15/32 (46%)	8/44 (18%)	.01
No. Patients with ≥4/5 Treatment response to Fludrocortisone	13/76 (17%)	11/32 (34%)	2/44 (4%)	.09

**Table 2:** Summary of the most common symptoms in each group.

Symptom	Overall *n* = 76	RGI *n* = 32	NRGI *n* = 44
Abdominal pain	43/76 (56%)	23/32 (71%)	20/44 (45%)
nausea	8/76 (10%)	5/32 (15%)	3/44 (6%)
dizziness	13/76 (17%)	3/32 (9%)	10/44 (22%)
vomiting	13/76 (17%)	4/32 (12%)	9/44 (20%)
syncope	6/76 (7%)	2/32 (6%)	4/44 (9%)
fatigue	2/76 (2%)	1/32 (3%)	1/44 (2%)
diarrhea	5/76 (6%)	1/32 (3%)	4/44 (9%)
headache	5/76 (6%)	1/32 (3%)	4/44 (9%)

**Table 3:** Treatment choices in both groups.

Treatment group	Overall	RGI *n* = 32	NRGI *n* = 44
Volume expansion/ Fludrocortisone	23/76 (30%)	15/32 (47%)	8/44 (18%)
Tricyclic antidepressant	9/76 (12%)	6/32 (19%)	3/44 (7%)
Beta-blockers	25/76 (32%)	10/32 (31%)	15/44 (34%)
Cyproheptadine	3/76 (4%)	2/32 (6%)	1/44 (2%)
SSRI's	18/76 (23%)	10/32 (31%)	8/44 (18%)
Antiepileptic	5/76 (6%)	2/32 (6%)	3/44 (7%)
Proamatine	9/76 (12%)	5/32 (16%)	4/44 (9%)
Neurontin	2/76 (2%)	1/32 (3%)	1/44 (2%)
Mestinon	1/76 (1%)	1/32 (3%)	0/44 (0%)
No Treatment	17/76 (22%)	3/32 (9%)	14/44 (31%)
